# Generation of human vascularized and chambered cardiac organoids for cardiac disease modelling and drug evaluation

**DOI:** 10.1111/cpr.13631

**Published:** 2024-03-07

**Authors:** Jingsi Yang, Wei Lei, Yang Xiao, Shuai Tan, Jiani Yang, Yingjiong Lin, Zhuangzhuang Yang, Dandan Zhao, Chunxiang Zhang, Zhenya Shen, Shijun Hu

**Affiliations:** ^1^ Department of Cardiovascular Surgery of the First Affiliated Hospital & Institute for Cardiovascular Science, Collaborative Innovation Center of Hematology, State Key Laboratory of Radiation Medicine and Protection Suzhou Medical College, Soochow University Suzhou China; ^2^ Department of Cardiology, Key Laboratory of Medical Electrophysiology, Ministry of Education, Institute of Cardiovascular Research, the Affiliated Hospital Southwest Medical University Luzhou China

## Abstract

Human induced pluripotent stem cell (hiPSC)‐derived cardiac organoids (COs) have shown great potential in modelling human heart development and cardiovascular diseases, a leading cause of global death. However, several limitations such as low reproducibility, limited vascularization and difficulty in formation of cardiac chamber were yet to be overcome. We established a new method for robust generation of COs, via combination of methodologies of hiPSC‐derived vascular spheres and directly differentiated cardiomyocytes from hiPSCs, and investigated the potential application of human COs in cardiac injury modelling and drug evaluation. The human COs we built displayed a vascularized and chamber‐like structure, and hence were named vaschamcardioids (vcCOs). These vcCOs exhibited approximately 90% spontaneous beating ratio. Single‐cell transcriptomics identified a total of six cell types in the vcCOs, including cardiomyocytes, cardiac precursor cells, endothelial cells, fibroblasts, etc. We successfully recaptured the processes of cardiac injury and fibrosis in vivo on vcCOs, and showed that the FDA‐approved medication captopril significantly attenuated cardiac injury‐induced fibrosis and functional disorders. In addition, the human vcCOs exhibited an obvious drug toxicity reaction to doxorubicin in a dose‐dependent manner. We developed a three‐step method for robust generation of chamber‐like and vascularized complex vcCOs, and our data suggested that vcCOs might become a useful model for understanding pathophysiological mechanisms of cardiovascular diseases, developing intervention strategies and screening drugs.

## Graphical abstract

Hu and colleagues developed a three‐step method for robust generation of chamber‐like and vascularized cardiac organoids, and demonstrated their potentials in cardiac disease modelling and drug screening.

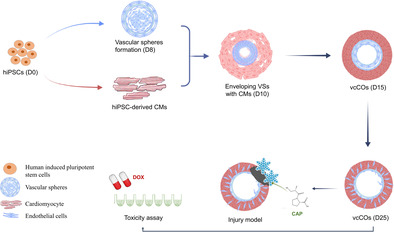

## 1 INTRODUCTION

Cardiovascular diseases (CVDs) are consistently ranked as the leading cause of global death, contributing to an estimated 19.05 million deaths in 2020 with an increase of 18.71% from 2010.^1^ Animal models of CVDs have been crucial in cardiac pathophysiological research and pharmaceutical development. Despite their advantages in disease modelling, genetic engineering, breeding and tissue accessibility, animal models have not been able to fully recapitulate human cardiac homeostasis and pathogenesis due to the huge interspecies differences, which led to a poor clinical translation of therapeutic entities and an extremely high drug attrition rate in clinical studies.^2^, ^3^ Therefore, human models are urgently needed for understanding the human‐specific cardiac pathologies and promoting the success rate of clinical treatment and drug development.

Since their discovery, human pluripotent stem cells (hPSCs), including human embryonic stem cells (hESCs) and human induced pluripotent stem cells (hiPSCs), have shown a great potential for in vitro modelling of human CVDs through robust differentiation into cardiac cell types.^4^ Human PSC‐derived cardiomyocytes (hPSC‐CMs) and endothelial cells (hPSC‐ECs) in two‐dimensional (2D) culture system have served as prevailing tools for pathological studies, drug screening and toxicity assessment in the cardiovascular field.^5^, ^6^, ^7^, ^8^ However, 2D hPSC‐CM and hPSC‐EC models have several limitations such as the lack of complex structure and multi‐cellular organization present in the living organ, and relative low cell maturity.

To overcome these limitations, the expectation of three‐dimensional (3D) culture models of hPSC‐derived cardiac lineage cells has been increasing.^4^, ^9^ Human cardiac organoids (hCOs) represent contracting cardiac spheroids formed by cardiac differentiation from self‐assembled aggregates of hPSCs or spherical microtissues formed by aggregation of pre‐differentiated cardiac lineage cells.^9^, ^10^ Distinct methods have been developed to generate hCOs recapitulating 3D morphogenesis and some key aspects of CVDs, which cannot be achieved by 2D cell culture methods.^9^, ^11^, ^12^, ^13^ Despite significant achievements, applications of hCOs in disease modelling, drug discovery and cardiac regenerative therapies are hampered by limitations yet to be overcome, including low reproducibility, limited vascularization, lack of nervous or immune system and missing other essential features of the heart.^5^, ^10^, ^14^

Here, we developed a highly reproducible methodology to generate chamber‐like and vascularized complex hCOs, called vaschamcardioids (vcCOs). The rationale for our protocol is envelopment of hiPSC‐derived and vascular fate‐determined aggregates with the differentiated hiPSC‐CMs to form new cardiac aggregates, so that the vascular committed cells could migrate outward in response to a VEGF gradient and vascularize the peripheral myocardium. In this study, we explored the single‐cell characterization of the vcCOs, and validated their potential application, which suggested that such vcCOs could be used as a promising platform for cardiac disease modelling, drug efficacy and safety studies.

## 2 MATERIALS AND METHODS

### 2.1 Human induced pluripotent stem cell culture

Human induced pluripotent stem cells used in this study were previously generated in our laboratory under the authorization of the Ethics Committee of Soochow University.^14^ The hiPSCs were routinely maintained in PSCeasy® medium (Cellapy, China) on Matrigel‐coated dishes, and were passaged at 80% confluence with 0.5 mM EDTA (Sigma‐Aldrich, United States). Thiazovivin (2 μM, Selleck Chemicals, United States), a rho‐associated protein kinase (ROCK) inhibitor, was added into the culture medium for 24 h to prevent dissociation‐induced apoptosis of hiPSCs. All cell cultures were maintained within a humidified incubator at 37°C, 5% CO_2_.

### 2.2 Cardiomyocyte differentiation

According to previous study, cardiomyocyte differentiation of hiPSCs was induced in the chemically defined medium (CDM3) consisting of RPMI 1640 medium (Thermo Fisher, United States), 500 μg/mL bovine serum albumin (BSA), (Sigma‐Aldrich, United States) and 213 μg/mL L‐ascorbic acid 2‐phosphate (Sigma‐Aldrich, United States).^5^ Briefly, hiPSCs at 95% confluence were cultured in CDM3 that successively supplemented with 5 μM CHIR99021 (Sigma‐Aldrich, United States) for 48 h, and 2 μM Wnt‐C59 (Selleck chemicals, United States) for 24 h. After Wnt‐C59 withdrawal, CDM3 was refreshed daily. Spontaneously beating cardiomyocytes were commonly observed from day 9 of cardiac differentiation. For cardiomyocyte purification, the cells were passaged onto gelatin‐coated dishes, and cultured in glucose‐free RPMI 1640 (Thermo Fisher, United States) supplemented with 500 μg/mL BSA, 213 μg/mL L‐ascorbic acid 2‐phosphate and 5 mM sodium DL‐lactate (Sigma‐Aldrich, United States) for 3 days.

### 2.3 Human vaschamcardioids generation

Vascular spheres were generated in a manner similar to that previously described.^15^ Briefly, hiPSCs were dissociated from matrigel‐coated dishes, resuspended with differentiation medium (DMEM:F12 medium, 20% KSR, Glutamax, NEAA; all from Gibco, United States) and cultured in Ultra‐Low Attachment 6‐well plates (Corning, Unite States) overnight. After aggregate formation in the presence of Y‐27632, hiPSCs were induced for vascular lineage cell differentiation by successive treatments with CHIR99021 (12 μM) for 2 days, and a cocktail of BMP4 (25 ng/mL, PeproTech, United States), FGF2 (25 ng/mL, Novoprotein, China) and VEGFA (50 ng/mL, STEMCELL, Canada) for 6 days. The differentiation medium was changed every second day. Each differentiated‐vascular sphere was then replated along with 1 × 10^5^ hiPSC‐derived cardiomyocytes into one well of an ultra‐low attachment 96‐well plate (Corning, Unite States), and cultured in Claycomb medium (Sigma‐Aldrich, United States) supplemented with 15% fetal bovine serum (FBS, Vivacell, New Zealand), FGF2 (100 ng/mL), VEGFA (100 ng/mL) and Thiazovivin. After aggregation of hiPSC‐CMs surrounding the vascular spheres, the newly formed spheroids were cultured in Claycomb medium supplemented with 15% FBS, VEGFA and FGF2 to induce migration of vascular committed cells into the peripheral myocardium, and final formation of vcCOs.

### 2.4 Whole‐mount staining

The human vcCOs were collected in microcentrifuge tubes (Eppendorf, Germany) and fixed in 4% paraformaldehyde (PFA) solution for 1 h. After washing with phosphate buffer saline (PBS) solution, human vcCOs were permeabilized and blocked in PBS solution with 10% FBS, 0.5% BSA and 0.5% Triton X‐100 for 2 h, followed by successive incubation with primary antibodies at room temperature for 24 h or at 4°C for 48 h and appropriate Alexa Fluor fluorescent secondary antibodies at room temperature for 24 h. After counterstaining with Hoechst 33342 (MCE, United States), the vcCOs were mounted on glass microscope slides (Fisher Scientific, Unite States) for imaging using Zeiss LSM 880 confocal microscope. All antibodies used for immunofluorescent staining are listed in Table S1.

### 2.5 Immunofluorescence staining on frozen sections

The vcCOs were fixed in 4% PFA for 1 h, after which the samples were dehydrated in a gradient of 15% and 30% sucrose, and then frozen in liquid nitrogen at the optimal cutting temperature (OCT) compound (Tissue‐Tek, Netherlands), and sliced into 7 μm‐thick cryostat sections. Sections were blocked with PBS containing 5% BSA and 3% FBS for 1 h, and were incubated with primary antibodies overnight at 4°C. After incubation with the appropriate secondary antibodies for 1 h at room temperature, sections were counterstained with Hoechst 33342. Finally, sections were imaged using fluorescent microscope or confocal microscope, and were analysed using ImageJ. All antibodies used for immunofluorescent staining are listed in Table S1.

### 2.6 Three‐dimensional imaging of whole‐organoid

The organoids were fixed with 4% PFA at 4°C overnight and then post‐fixed at room temperature for 1 h. The iDISCO method as described online (https://idisco.info/) was used for immunofluorescent staining and deep clearing of whole‐organoids. After increasing the optical transparency with 100% dibenzyl ether, organoids were placed in a chamber filled with dibenzyl ether and were imaged by using the LiTone XL light‐sheet microscope (Light Innovation Technology, China). After setting the four 3D scanning sides (anterior, posterior, left and right) of the organoids, 4× and 10× objective lens was used to scan. The 405,488 and 594 lasers were selected for three‐band scanning of the whole organoids. The LiT Software was used to process the obtained raw images. For detailed analysis, we used the Bitplane Imaris software 9.0.1 (Oxford Instruments, UK) to perform 3D reconstruction and 3D data analysis of the images obtained by iDISCO and CLARITY methods. Several algorithms in the Imaris software were used including 3D viwer, and surface.

### 2.7 Quantitative real‐time polymerase chain reaction

Total RNAs were extracted from different samples using TRI reagent (Thermo Fisher, United States), and were subjected to reverse transcription for single‐strand cDNA synthesis using a Takara Prime Script RT Reagent Kit (Takara, Japan). Quantitative real‐time polymerase chain reaction (qPCR) assays were performed using an Applied Biosystems StepOnePlus Real‐Time PCR System (Thermo Fisher, United States). The 18S rRNA was used as a reference gene. The data were analysed using the 2^−ΔΔCT^ method. All primers used for qPCR assays are listed in Table S2.

### 2.8 Single‐cell suspension preparation

The cells in human vcCOs were dissociated in the digestion solution (0.25% trypsin +0.1% collagenase +0.5 mM EDTA) for 15 min at 37°C. After centrifugation at 300 *g* for 3 min, cells were resuspended with resuspension solution (0.04% BSA + 0.01 mM EDTA), followed by filtration with 40 μm filters. The Countess® II Automated Cell Counter (Thermo Fisher, United States) was used to calculate the proportion of living cells. Cell suspensions at a concentration of 1, 000 cells/μL and ≥90% viability were used for single‐cell RNA sequencing (scRNA‐seq) analysis.

### 2.9 Single‐cell RNA sequencing

Cellular suspensions were loaded on a 10× Genomics GemCode single‐cell instrument to generate single‐cell Gel Beads‐In‐Emulsion (GEMs). The scRNA‐seq libraries were prepared using Chromium Next GEM Single Cell 3' Reagent Kits v3.1 according to manufacturer's protocol. Illumina sequencing platform was used for high‐throughput sequencing of libraries. The 10× Genomics Cell Ranger software (version 3.1.0) was used for conversion of raw BCL files to FASTQ files, alignment and counts quantification. The cell by gene matrices for each sample were individually imported to Seurat version 4.4.0 for downstream analysis. Integrated expression matrix is then scaled and performed on principal component analysis (PCA) for dimensional reduction. For visualization of clusters, t‐distributed Stochastic Neighbor Embedding (t‐SNE) was generated using the same PCs. The log‐normalized matrices were then loaded on SingleR packages for cell type annotation, which was based on correlating gene expression of reference cell types with single‐cell expression. GO enrichment analysis provides all GO terms that significantly enriched in differentially expressed genes comparing to the genome background and filter the differentially expressed genes that correspond to biological functions. All peak related genes were mapped to GO terms in the Gene Ontology database (http://www.geneontology.org/). The calculated *p*‐value went through FDR Correction, taking FDR ≤0.05 as a threshold. KEGG is the major public pathway‐related database. Pathway enrichment analysis identified significantly enriched metabolic pathways or signal transduction pathways in differentially expressed genes comparing with the whole genome background. The organoid data were integrated and compared with publicly available scRNA‐seq datasets of human adult (GSE109816) and fetal (GSE106118) hearts.

### 2.10 Flow cytometric analysis

After digestion, cells were filtered through a 70 μm nylon mesh (Fisher Scientific, USA) to remove cell clumps, and subjected to direct or indirect immunostaining for the markers of cardiomyocytes (cTnT), endothelial cells (PECAM1) and fibroblasts (COL1A2), respectively. Flow cytometric analysis was performed using a Guava EasyCyte™ 8 flow cytometer (EMD Millipore, Germany). Data were analysed with FlowJo software. The antibodies used for flow cytometry are listed in Table S1.

### 2.11 Cryoinjury of human vcCOs and captopril treatment

Cryoinjury was performed on vcCOs and cardiomyocyte spheres using a sterile syringe needle precooled with the liquid N_2_. Prior to cryoinjury, culture medium was removed, so that the needle tip was able to precise contact with the vcCOs and CM spheres for 3 s. Following cryoinjury, the vcCOs and CM spheres were immediately supplemented with culture medium with or without captopril (20 mM, Sigma‐Aldrich, United States), and cultured for 3 days until subsequent analysis.

### 2.12 Calcium transient assay

The vcCOs were loaded with 4 μM Fluo 4‐AM (Thermo Fisher, United States) at 37°C for 40 min. After washing with PBS, the vcCOs were mounted in glass‐bottom dishes with Tyrode's solution and observed under a fluorescence microscope. The green fluorescent signals were monitored and analysed to calculate the amplitude of the Ca^2+^ transient (∆F/F0). The time to peak was recorded. All of these measurements were acquired for at least four beats in each video and averaged for comparison.

### 2.13 Enzyme‐linked immunosorbent assay

The cTnT levels in culture supernatant from vcCOs and CM spheres were measured by using commercially available kits based on enzyme‐linked immunosorbent assay methods (Cusabio Tenascin, China). The data and standard curves were analysed by cvxpt32 software.

### 2.14 TUNEL assay

The cryostat sections were fixed with 4% PFA for 30 min and permeabilized with 0.1% Triton X‐100 in pre‐cold PBS for 2 min. Sections were then incubated with 50 μL TUNEL assay solution according to manufacturer's instructions (In Situ Cell Death Det Kit, TMR red; Roche, Germany), protected from light, for 30 min at 37°C. After nuclear staining with Hoechst 33342, the sections were observed and photographed under a fluorescence microscope.

### 2.15 Quantitative cytotoxicity assays

The vcCOs were incubated with different doses of doxorubicin (0.1, 1, 10 and 50 μM) for 24, 48, or 72 h, respectively. The cell viability, ATP production and apoptosis were evaluated by Cell Counting Kit 8 (CCK8) Assay (Dojindo, Japan), CellTiter‐Glo Viability Assay (Promega, USA) and Annexin V‐FITC Apoptosis Detection kit (Vazyme, China), respectively, according to the manufacturer‐recommended procedures.

### 2.16 Statistical analysis

Comparisons between two groups were performed using Student's *t*‐test. Comparisons among multiple groups were performed with one‐way analysis of variance (ANOVA). Statistical significance was denoted by a *p* < 0.05. All data were presented as the mean ± SEM.

## 3 RESULTS

### 3.1 Generation of chambered and vascularized vcCOs from hiPSCs

**FIGURE 1:**
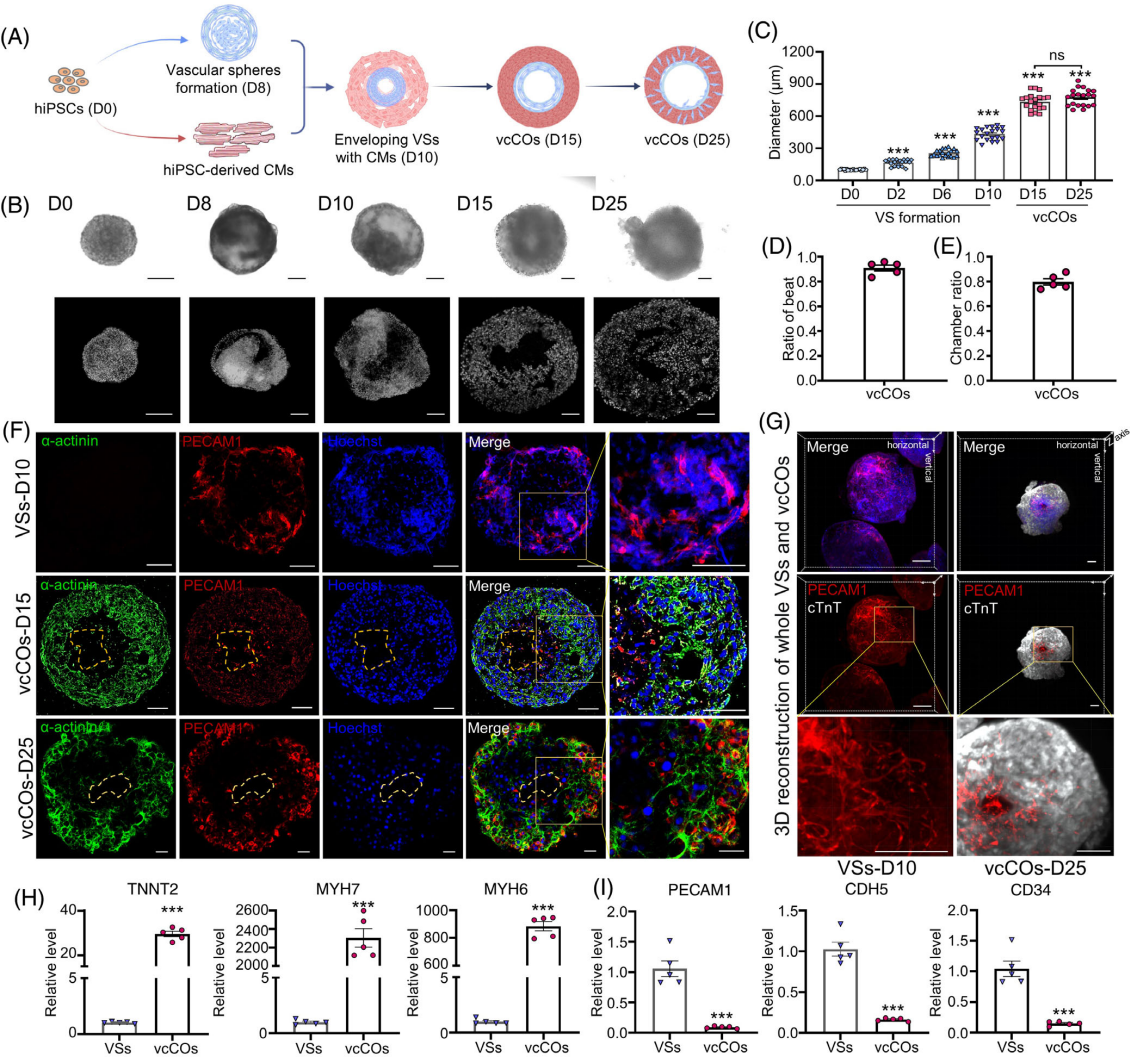
Generation and characterization of vaschamcardioids (vcCOs) with endothelial cells from human induced pluripotent stem cell (hiPSCs). (A) Schematic diagram of the 3D culture method for generation of chambered cardiac organoids with vascular system. (B) Representative brightfield and nuclear staining images of hiPSC‐derived spheres at different stages during organoid formation. Scale bar indicates 100 μm. (C) Diameter of the spheres at different stages. (D) Ratio of vcCOs showing spontaneous beating at day 15 (*n* = 5). (E) Ratio of vcCOs with a chamber (*n* = 5). (F) Whole‐mount staining of vascular spheres (VSs) and cardiac organoids for endothelial cells (PECAM1, red), cardiomyocytes (α‐actinin, green) and the nucleus (Hoechst 33342, blue) at indicated days (D10, D15 and D25). Scale bar indicates 50 μm. (G) 3D reconstruction of whole VSs (D10) and vcCOs (D25) with light plate microscope. Different components highlighted in (G) are colour‐coded throughout all the images (PECAM1, red; cTnT, White; Hoechst 33342, blue), scale bar indicates 100 μm. (H, I) Real‐time PCR analysis of mRNA levels of cardiac (TNNT2, MYH7, MYH6) and endothelial (PECAM1, CHD5, CD34) specific markers in vcCOs on D25 (*n* = 5). Student's *t*‐test; *p < 0.05, ***p < 0.001 and ns, not significant.

To improve vascularization and chambering properties of human COs, as well as reproducibility, we designed a three‐step method as shown in Figure 1A: (1) induction of hiPSC aggregates to gradually differentiate towards the mesoderm and subsequent vascular cell fate to form the vascular spheres; (2) enveloping each of the vascular spheres with 1 × 10^5^ purified and beating hiPSC‐CMs to form new spheres; (3) inducing out‐migration of central vascular cells in response to a VEGF gradient caused by cardiomyocyte encapsulation, and vascularization of the peripheral myocardium. This protocol allows the derivation of beating COs in 2 days post‐hiPSC‐CMs envelopment, and the beating generally stabilizes after 5 days, which corresponds to 15 days from initiation of hiPSC differentiation (Figure 1B, Movie S1).

According to the brightfield images, we noticed obvious morphological changes, such as gradually increased sphere size and complexity, in the formed aggregates during vascular lineage cell induction and the envelopment of hiPSC‐CMs (Figure 1B,C). Meanwhile, nuclear staining images revealed distinct chambers in the aggregates from day 8 onwards during the process of organoid generation (Figure 1B). During maintenance culture, the aggregate volume was maintained at a stable level (Figure 1B,C). The ratio of beating COs at day 15 is more than 90% (Figure 1D **)**. About 80% COs showed a chamber‐like structure in the sphere (Figure 1E). We performed confocal microscopy for α‐actinin and PECAM1 to visualize the location of cardiomyocytes and endothelial cells, respectively. Confocal imaging results revealed the presence of a chamber in the centre of constructed human COs, which were surrounded by α‐actinin‐positive cardiomyocytes and PECAM1‐positive endothelial cells (Figure 1F). When COs at day 15 of differentiation showed a clustered distribution of endothelial cells in the inner area, more endothelial cells accumulated towards the outer area and became embedded in the myocardium at day 25 (Figure 1F). We further validated the presences of PDGFRβ‐positive pericytes and α‐SMA‐positive smooth muscle cells in COs, while some of these cells localized adjacent to PECAM1‐positive endothelial cells (Figure S1). To get a better look at the 3D structure of organoids, we used the immunolabelling‐enabled 3D imaging of solysoly‐cleared organs (iDISCO) method to make organoids transparent. A panoramic scan was then taken under a light plate microscope. It can be clearly seen that vascular spheres on day 10 have obvious generation of vascular network, while vcCOs have obvious and ordered vascular network and spreads from inside to outside on day 25 (Figure 1G). The qPCR results also showed abundant expression of marker genes of cardiomyocytes (TNNT2, MYH7 and MYH6), and the expression levels of marker endothelial cells (PECAM1, CDH5 and CD34) were relatively lower in vcCOs on day 25 when compared to that in vascular spheres (Figure 1H,I).

Taken together, the hiPSC‐derived cardiac organoids generated by our protocol were characterized by central chambers and vascularization. Thus, we named the generated COs as vaschamcardioids (vascularized‐ and chambered‐cardiac organoids, vcCOs). These vcCOs keep beating spontaneously for at least 8 weeks in culture medium, corresponding to the longest time tested in our study (Movie S2).

### 3.2 Cellular composition analysis of vcCOs by single‐cell transcriptomics

**FIGURE 2:**
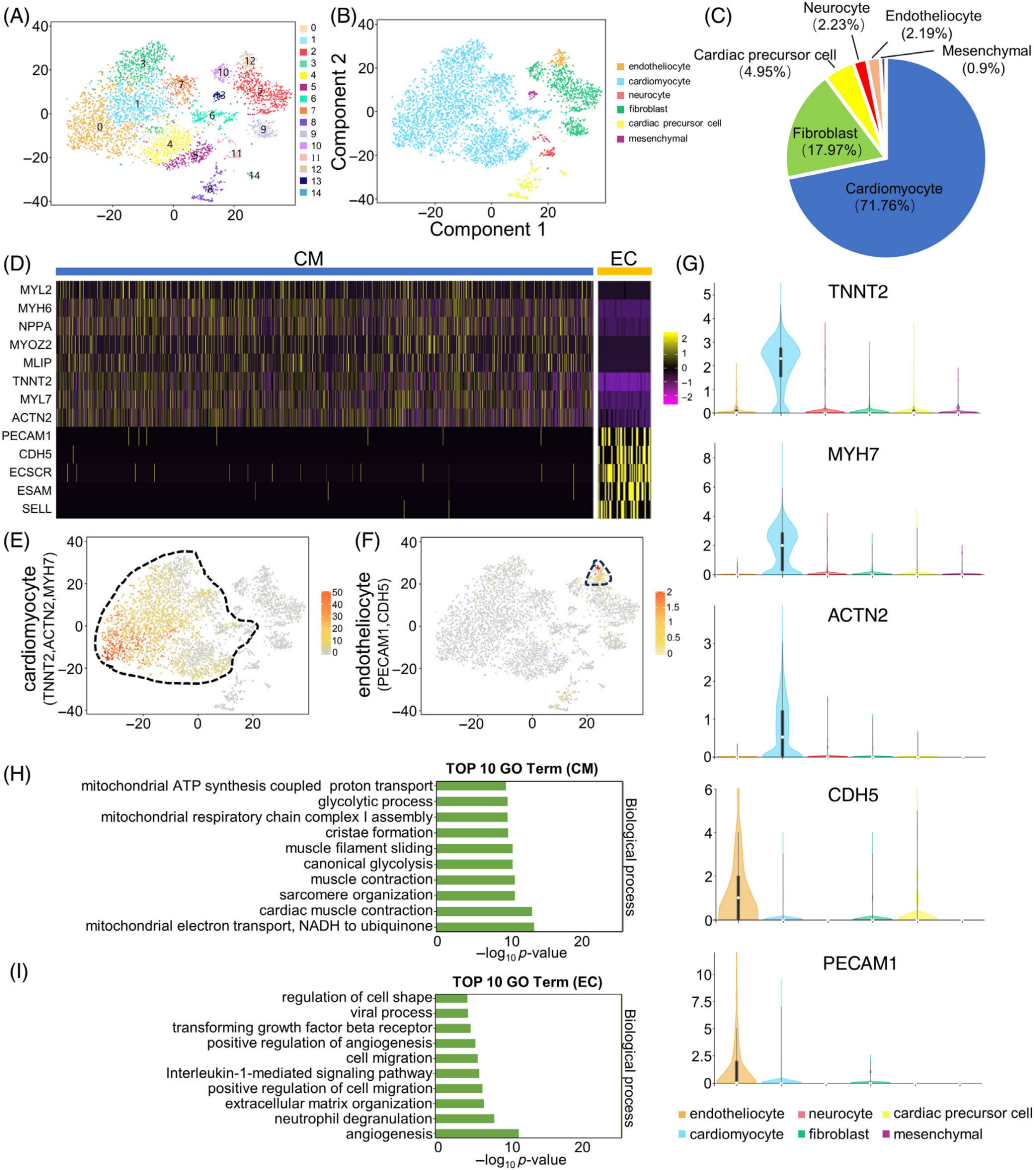
Single‐cell RNA‐Seq analyses of human vaschamcardioids (vcCOs) with multiple cardiac‐specific cell lineages. (A) t‐distributed Stochastic Neighbor Embedding (t‐SNE) visualization of clustering based on transcriptome similarities revealing 15 cell clusters in vcCOs. (B) t‐SNE representation showing different cell types, coloured according to the signature gene expression of different cells in the heart (cardiomyocytes, endothelial cells, cardiac progenitor cells, fibroblasts, neurocyte and mesenchymal). (C) The proportion of multilineage organoids corresponding to different clusters. (D) Heatmap of top marker genes of cardiomyocyte cluster (CM) and endotheliocyte cluster (EC). (E, F) t‐SNE representation showing expression of marker genes in the cardiomyocyte cluster and endotheliocyte cluster. (G) Violin plots of signature genes confirming the cardiomyocyte cluster and endotheliocyte cluster. (H, I) gene ontology (GO) analysis showing the enriched biological processes according to the signature genes in the cardiomyocyte cluster and endotheliocyte cluster. An FDR‐adjusted *p*‐value of 0.05 was set as a threshold.

To assess cellular composition of vcCOs, we performed scRNA‐seq analysis of vcCOs at day 25. The t‐SNE plots of the aggregated scRNA‐seq data were automatically divided into 15 groups in vcCOs, which can be classified into six cell types according to the gene expression signature of different cells in the heart (Figure 2A,B). The identified cell types include cardiomyocytes (CMs), endothelial cells (ECs), fibroblasts (FBs), cardiac progenitor cells (CPCs), neurocytes (NCs) and mesenchymal cells (MSCs). Among them, cardiomyocytes were the most abundant type of cells, accounting for about 71.76% of all living cells, while fibroblasts and endothelial cells accounted for 17.97% and 2.19%, respectively (Figure 2B,C). Flow cytometric analysis identified a consistent proportion of cardiomyocytes (64.7 ± 6%) in vcCOs, but a relatively higher proportion of endothelial cells (6.4% ± 2%) and a lower proportion of fibroblasts (10.7% ± 3%) compared to single‐cell analysis (Figure S2A–C), which was probably due to varying detection sensitivities. Nevertheless, these data indicated that the vcCOs were composed of the typical heart‐developing cell types.

When focusing on cardiomyocytes and endothelial cells, the two most important cell types in heart, we generated a heatmap showing cluster analyses of the differentially expressed genes (DEGs) to represent the transcriptomic profile of these two cell types (Figure 2D). While most cardiomyocytes in vcCOs showed cell‐specific expression of TNNT2, MYH7 and ACTN2 (Figure 2D,E), abundant expressions of CDH5 and PECAM1 were detected in the endotheliocyte cluster (Figure 2D,F), which were also demonstrated by violin diagrams (Figure 2G). We then performed Gene Ontology (GO) analysis of DEGs in each cluster. The GO terms involving sarcomere organization, muscle contraction and mitochondrial function were enriched in the cardiomyocyte cluster (Figures 2H and S3A), while DEGs in the endotheliocyte cluster were mainly related to angiogenesis, neutrophil degranulation and extracellular matrix organization (Figures 2I and S3B). Meanwhile, we compared the scRNA‐seq data of vcCOs with human fetal and adult hearts, revealing a relatively intermediate status of the cardiomyocytes in vcCOs, which, however, was closer to cardiomyocytes in adult hearts (Figure S4). Nevertheless, these vcCOs present a promising in vitro model for exploring human heart development and cardiac regeneration.

Taken together, the above data confirmed the multicellular cell type composition of human vcCOs, indicating that cardiac lineage cells, especially cardiomyocytes, were the major cell type composing this kind of cardiac organoid.

### 3.3 Cardiomyocyte‐endothelial cell interaction analysis in human vcCOs

**FIGURE 3:**
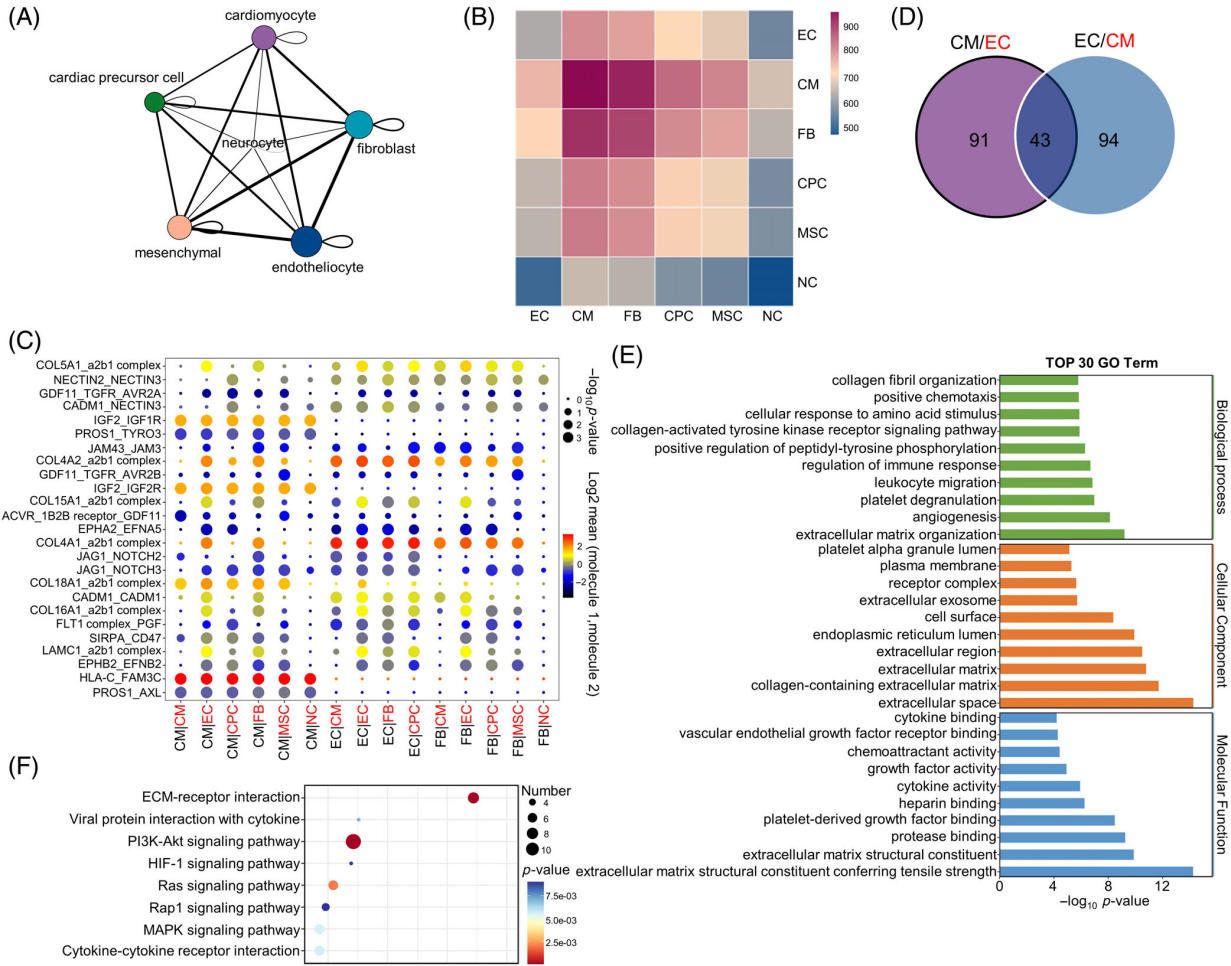
Cardiomyocyte–endothelial cell interaction analysis in human vaschamcardioids (vcCOs). (A) Inferred interaction networks between different cell populations based on ligand–receptor pair analysis of scRNA‐seq data. The edge width of the line is proportional to the number of specified ligand–receptor pairs, and the size of the circle is proportional to the number of units in each cell group. (B) Heatmap showing significant ligand–receptor interactions between different cluster (*p*‐value < 0.05, permutation test). The protein interactions are extrapolated according to their mRNA levels. (C) Overview of top 25 ligand–receptor interactions between different cell types. X‐axis and Y‐axis represent cell–cell pair and ligand–receptor pair, respectively. Each bubble represents a ligand–receptor pair in a cell–cell pair. (D) Venn diagram showing overlap of the ligand–receptor pairs between the cardiomyocyte‐to‐endotheliocyte (CM/EC) and endotheliocyte‐to‐cardiomyocyte (EC/CM) orientations. (E) Gene ontology (GO) analysis of the 43 overlapped ligand–receptor pairs in biological process, cellular component and molecular function. (F) KEGG pathways associated with the overlapped ligand–receptor pairs. An FDR‐adjusted *p*‐value of 0.05 was set as a threshold.

To investigate the context‐dependent crosstalk of different cell types in vcCOs, we performed cell communication analysis based on the single‐cell gene expression matrix. Significant ligand–receptor pair interaction networks were inferred between different cell populations (Figure 3A,B). While both cardiomyocytes and fibroblasts showed close connection with most other cell components other than neurocytes, endothelial cells showed specific and strong connection with cardiomyocytes (Figure 3B). Meanwhile, among the total top 25 identified ligand–receptor pairs, both CM‐EC and EC‐CM ligand–receptor pairs were abundantly expressed in vcCOs (e.g. COL4A2_a2b1 complex, COL4A1_a2b1 complex, CADM1_CADM1 and so on). However, only few ligand–receptor pairs in the FB‐CM orientation were abundantly expressed in vcCOs (Figure 3C). Consistently, the Venn diagram demonstrated an overlap of 43 significant ligand–receptor pairs between the CM‐EC and EC‐CM orientations, but only eight significant ligand–receptor pairs between the CM‐FB and FB‐CM orientations (Figures 3D and S5). Therefore, we focused on the communication between cardiomyocytes and endothelial cells, and the 43 overlapped ligand–receptor pairs were subjected to subsequent GO and KEGG analysis.

GO analysis showed that the overlapped ligand–receptor pairs between CM‐EC and EC‐CM orientations were mainly related to the biological processes and molecular regulation related to angiogenesis and extracellular matrix tissue (Figure 3E). Meanwhile, KEGG analysis indicated that the PI3K‐AKT signalling pathway, ECM‐receptor interaction signalling pathway and Ras signalling pathway were significantly up‐regulated in myocardial and endothelial interactions (Figure 3F). Therefore, we speculated that above three signalling pathways were important for cell communication between cardiomyocytes and endotheliocytes in vcCOs.

### 3.4 Multi‐lineage human vcCOs simulate myocardial injury‐induced fibrosis

**FIGURE 4:**
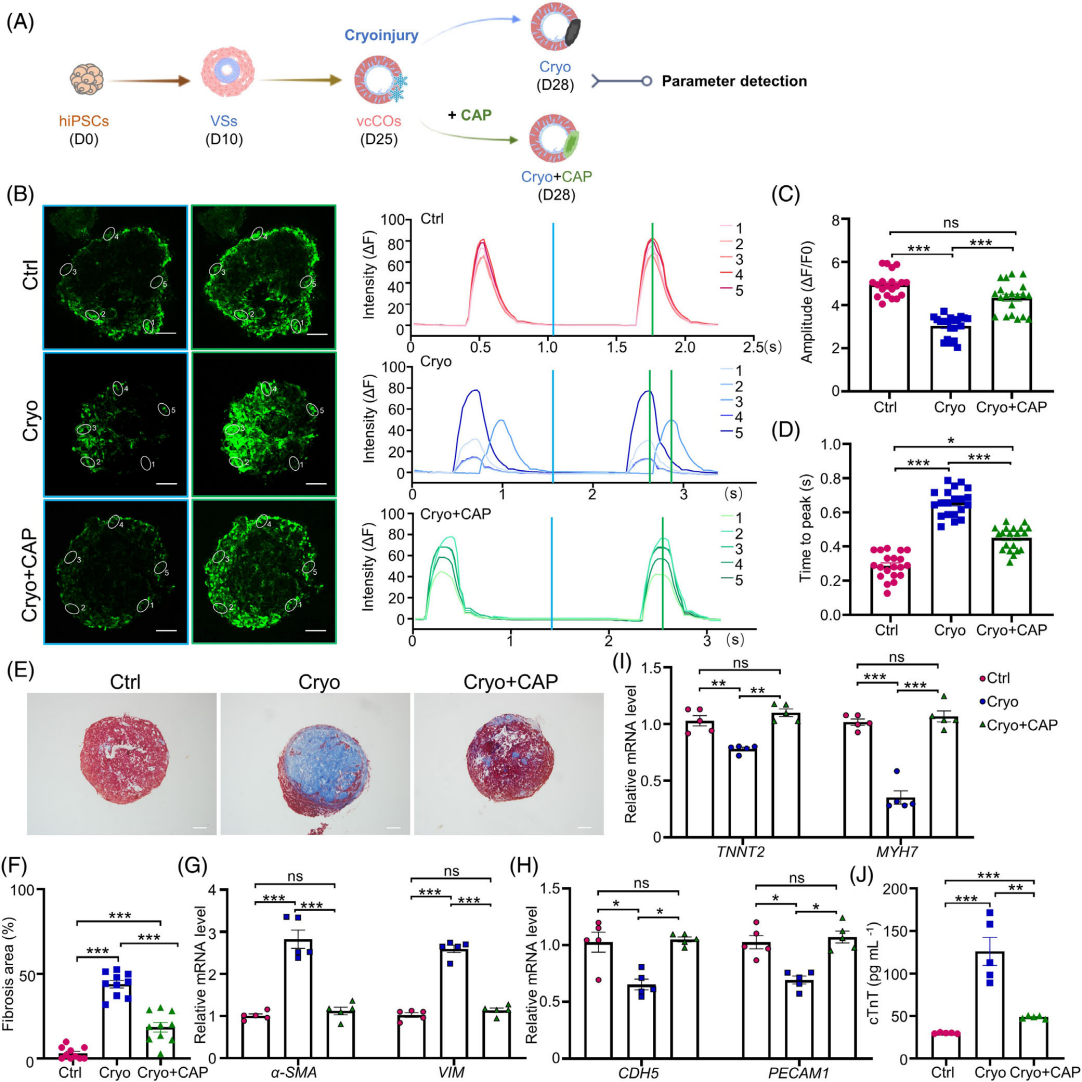
Multi‐lineage human vaschamcardioids (vcCOs) simulate myocardial injury‐induced fibrosis. (A) Schematic diagram for modelling and evaluating cryo‐induced injury in vcCOs. (B) Consistency assay of calcium transient in different regions of vcCOs with/without cryoinjury and captopril (CAP) treatment (*n* = 5). Representative images showing the time‐lapse records of calcium activity and the calcium handling traces in vcCOs from control (Ctrl, red trace), cryoinjury (Cryo, blue trace) and cryoinjury plus CAP treatment (Cryo+CAP, green trace) groups. The vcCOs were preloaded with the calcium indicator Fluo‐4 AM before image capture under microscope. (C, D) Ca^2+^ transient properties of vcCOs in control, cryoinjury and recovery groups, including the amplitude of Ca^2+^ transient (C) and the time to peak (D). (E) Masson's trichrome staining (blue, connective tissue; red, muscle) of cryo‐injured and recovered vcCOs in 3 days post‐injury. Scale bar, 100 μm. (F) Quantification of fibrotic areas. (G–I) Real‐time PCR analysis of fibroblast (*α*‐SMA, VIM), endothelial (CDH5, PECAM1) and myocardial (TNNT2, MYH7) marker genes in different vcCOs (*n* = 5). (J) Evaluation of cTnT levels in the culture medium by ELISA (*n* = 10). One‐way ANOVA; *p < 0.05, **p < 0.01, ***p < 0.001; ns, not significant.

To explore the potential of human vcCOs in disease modelling, we performed cryoinjury of vcCOs to simulate the process of myocardial infarction (MI) in humans. Cryoinjury was introduced at day 25 of hiPSC differentiation into vcCOs, and calcium transients were recorded at day 28 to assess changes in functional characteristics of vcCOs (Figure 4A). At the same time, we attended to further investigate whether captopril (CAP), an FDA‐approved medication used in the management of MI, could relieve functional disorders in cryo‐injured vcCOs.

We found that, compared with the control group, cryoinjury led to asynchronous contraction (Movies S3 and S4) and calcium transient recordings (Figure 4B) in cardiomyocytes from different regions of human vcCOs. Meanwhile, calcium transient parameter analysis indicated a significant reduction in calcium handling capacity of cryo‐injured vcCOs, as evidenced by a decrease in peak amplitude and an increase in time to peak (Figure 4C,D). However, CAP treatment could amend asynchronous contraction, and promote the recovery of vcCOs from cryo‐induced impairment of calcium handling (Figure 4B,D, Movie S5). Analysis of Masson's trichrome‐stained vcCOs images demonstrated obvious fibrosis in cryo‐injured vcCOs, which were significantly prevented by CAP treatment (Figure 4E,F). Compared to the control group, cryo‐injured vcCOs showed a significantly increased expression of fibrotic marker genes such as α‐SMA and Vimentin (VIM), and a significantly decreased expression of both endothelial (CDH5, PECAM1) and myocardial (TNNT2, MYH7) marker genes (Figure 4G–I). Likewise, the CAP treatment effectively inhibited the fibrotic marker gene expression and promoted the expression of endothelial and myocardial marker genes in cryo‐injured vcCOs (Figure 4G–I), indicating a protective role of CAP in cryoinjury‐induced myocardial injury and fibrosis. We further evaluated the cTnT levels in the culture medium, a well‐established serum marker for acute cardiac injury, demonstrating a significant increase in the release of cTnT from cryo‐injured vcCOs and an inhibitory effect of CAP on cTnT release (Figure 4J).

Similarly, cryoinjury could also mimic acute cardiac injury on cardiomyocyte spheres lacking other cell components, as evidenced by fibrosis formation and cTnT release (Figure S6A–C). However, Masson staining results demonstrated a reduced degree of fibrosis in cardiomyocyte spheres post‐cryoinjury treatment, while CAP treatment failed to protect the cardiomyocyte spheres against cryoinjury‐induced fibrosis (Figure S6A,B). These data indicated that vcCOs are more suitable for simulating the pathological process of MI and testing the efficacy of preclinical drugs.

### 3.5 Multi‐lineage human vcCOs as a platform for drug toxicity assay

**FIGURE 5:**
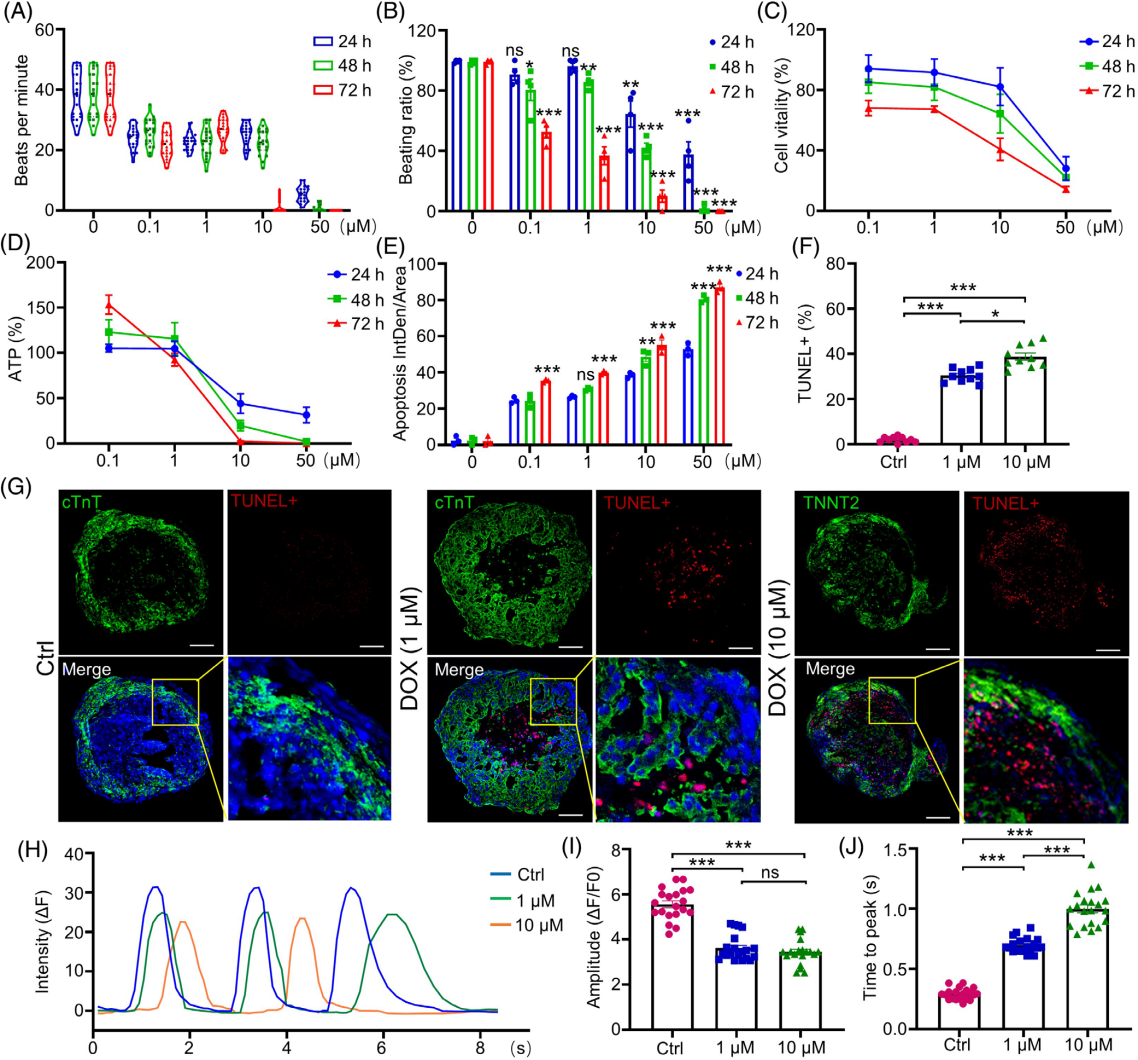
Multi‐lineage human vaschamcardioids (vcCOs) as a platform for drug toxicity assay. (A, B) Dose‐ and time‐dependent effects of doxorubicin (DOX) on beating frequency in vcCOs (A) and the ratio of beating organoids (B). Cardiac organoids were incubated with different doses of DOX (0, 0.1, 1, 10 and 50 μM) for 24, 48 and 72 h, respectively (*n* = 5). In contrast to 0 h, *p < 0.05, **p < 0.01, ***p < 0.001 and ns, not significant. (C) CCK8 assay results for vcCOs after a 24, 48 or 72 h treatment with DOX (*n* = 5). (D) ATP levels in vcCOs after doxorubicin treatments (*n* = 5). (E) Quantitative analysis of fluorescence intensity following Annexin V‐FITC staining in multi‐lineage human vcCOs (*n* = 5). In contrast to 24 h of the same concentration, *p < 0.05, **p < 0.01, ***p < 0.001 and ns, not significant. (F, G) Apoptotic staining (TUNEL) of organoid sections after exposure to DOX for 48 h at the indicated concentrations. (H) Representative images showing the time‐lapse recording of calcium activity and the calcium handling traces from control (Ctrl, blue trace) and DOX‐treated (green and yellow trace) vcCOs preloaded with the calcium indicator Fluo‐4 AM. (I, J) Ca^2+^ transient properties of control and DOX‐treated vcCOs, including amplitude of Ca^2+^ transient (I), the time to peak (J). One‐way ANOVA; *p < 0.05, **p < 0.01, ***p < 0.001, and ns, not significant.

Organoids are also an emerging choice for drug toxicity testing and potential drug screening. In this study, we chose doxorubicin (DOX), an effective and frequently used chemotherapeutic drug with cardiotoxicity, to assess the feasibility of vcCOs as a platform for drug toxicity analysis. Our data indicated DOX treatments significantly reduced the beating frequency in vcCOs in a dose‐ and time‐dependent manner (Figure 5A), while high doses of doxorubicin, such as 10 μM treatment for 72 h and 50 μM treatment for 48 h, could almost completely inhibit the beating of human vcCOs (Figure 5A,B). Cell viability in vcCOs was then evaluated by CCK8 and cellular ATP content assays, and showed a dose‐ and time‐dependent inhibition by doxorubicin treatment (Figure 5C,D). Meanwhile, we detected cell apoptosis in DOX‐treated organoids by quantitative analysis of the fluorescence intensities following Annexin V‐FITC staining. Our data indicated a dose‐ and time‐dependent increase of cell apoptosis in vcCOs in response to doxorubicin (Figure 5E), which was consistent with data from TUNEL assay showing increased TUNEL‐positive cells in vcCOs treated with high concentration of doxorubicin (Figure 5F,G).

To assess the influence of DOX in the contractibility of human vcCOs, we performed calcium transient assay on these organoids after DOX treatment for 48 h. Compared to the control group, the vcCOs treated with either 1 or 10 μM of DOX showed a significant decrease in the peak amplitude and an increase in the time to peak, indicating an impaired capacity of calcium handling in these vcCOs (Figure 5H–J). Taken together, the human vcCOs we built provided a suitable in vitro model for cardiac toxicity assay of preclinical drugs.

## 4 DISCUSSION

Considerable progress has been made in developing laboratory models of human development and diseases over the past decades, starting with animal models and primary cell culture, and moving onwards to hPSC‐derived functional cells, engineering tissues and organoids.^16^, ^17^ Despite the great potential in understanding human cardiovascular disorders for treatment and prevention, research progress of human COs lags significantly behind other organs such as brain, kidney and intestine.^18^, ^19^, ^20^ Recently, methodologies to create COs from hPSCs have been emerging.^10^, ^21^, ^22^, ^23^, ^24^ However, limitations such as low reproducibility, limited vascularization and lack of chamber specificity still need to be addressed for the COs.^25^ In this study, we developed a three‐step method for robust generation of chamber‐like and vascularized complex vcCOs, which were suitable for cardiac disease modelling and drug evaluation.

In our method, hiPSC‐derived self‐assembling vascular spheres were encapsulated with cardiomyocytes directly differentiated from hiPSCs, followed by induction of out‐migration of central vascular cells. The vascular spheres were generated partially according to previous methods for blood vessel organoids from hiPSCs, in which the vascular spheres were embedded in a collagen I‐Matrigel matrix to induce vessel sprouting and mature into stable blood vessels.^15^, ^26^ Instead, the collagen I‐Matrigel matrix was replaced by hiPSC‐derived cardiomyocytes in our method. The use of directly differentiated and purified hiPSC‐CMs ensured the success rate of vcCOs, resulting in approximately 90% of vcCOs beating spontaneously in 2 days post‐encapsulation. Meanwhile, the surrounding cardiomyocytes layer could serve as a scaffold for the outward migration of vascular cells in response to a VEGF gradient, leading to the formation of vascularized and chambered human COs. These chamber‐like and vascularized multicellular COs may better mimic heart structure than microtissues formed by aggregation of pre‐differentiated cardiac lineage cells.^9^, ^27^, ^28^

In the adult human heart, cardiomyocytes constitute approximately 30.1% to 49.2% of the total cell number.^9^, ^29^ In contrast, they make up a higher proportion, ranging from 65% to 80%, of total cardiac cells in the fetal heart.^30^, ^31^, ^32^ The proportion of cardiomyocytes in our vcCOs is similar to that in fetal heart. Probably due to varying detection sensitivities, flow cytometry identified a relatively higher proportion of endothelial cells and a lower proportion of fibroblasts compared to single‐cell analysis. Interestingly, the proportion of endothelial cells in vcCOs, as detected by flow cytometry, aligns with previous studies in adult and fetal human hearts.^29^, ^30^, ^32^, ^33^ Through comparative analysis with the scRNA‐seq data of adult/fetal hearts,^30^, ^34^ we found that the status of cardiomyocytes in our vcCOs lies between the fetal and adult hearts, and was more skewed towards the adult heart. To better mimic the adult heart, follow‐up studies should focus on regulating the proportion and maturity of cardiomyocytes in vcCOs.

Our data indicated a strong ligand–receptor communication between endothelial cells and cardiomyocytes in human vcCOs, via PI3K‐AKT, ECM receptor interaction and Ras signalling and other pathways. PI3K‐AKT pathway plays an important role in promoting the growth and survival of cardiomyocytes, boosting angiogenesis and maintaining cardiac systolic function during cardiac development.^35^, ^36^ Moreover, PI3K‐AKT pathway can promote the migration of endothelial cells and the formation of myocardial capillaries under the stimulation of VEGF.^37^, ^38^ At the same time, ECM signalling pathway not only plays an important role in cardiac remodelling, but also affects cell differentiation, proliferation and migration.^39^, ^40^ Ras signalling pathway is also very classical, and it often works with AngII to regulate cardiomyocyte proliferation and conduction, as well as endothelial cell adhesion and blood vessel formation during cardiac development.^41^, ^42^ The above content indicates the potential roles of cardiomyocyte–endothelial cell communication in vascularization of cardiac organoids and heart development.

The development of most CVDs was accompanied by a complex cardiac remodelling process involving multiple cell types and cell–cell interactions. Thus, multicellular composition and cellular phenotype are important for modelling cardiac diseases with COs. Both endothelial cells and fibroblasts have been shown to improve cardiomyocyte maturation in COs and are critical for cardiac disease modelling.^9^, ^43^ With the presence of endothelial cells and fibroblasts, we were able to explore the process of cardiac fibrosis and repair in vcCOs. Compared to hypoxia treatment, cryoinjury induced cardiomyocyte death in a localized area, thus more closely recapitulates cardiac injury in MI patients showing cell death in only a particular region of the heart.^44^ Meanwhile, cryoinjury, as a simple and precise method for inducing localized cell death, has been widely used in lower vertebrates and neonatal rodents to study cardiac regeneration.^45^ Hence, cryoinjury was deliberately selected as the preferred method for modelling MI in the miniature vcCOs. Our data demonstrated a successful induction of cardiac injury and fibrosis in vcCOs, along with a protective role of the FDA‐approved medication captopril. These findings align with data from clinical trials and animal models.^46^, ^47^

In comparison with vcCOs, cardiomyocyte spheres lacking of other cell components developed a reduced degree of fibrosis after cryoinjury treatment. Importantly, CAP treatment failed to protect the cardiomyocyte spheres against cryoinjury‐induced fibrosis. These findings suggest that other components in the vcCOs, such as endothelial cells and fibroblasts, may play critical roles in cryoinjury‐induced fibrosis and in response to CAP treatment. Consequently, vcCOs are more suitable for simulating the pathological process of MI. However, a limitation of our vcCOs is the lack of immune cells which are known to play an important role in the early response post‐MI and contribute to the activation of the subsequent fibrotic response.^24^, ^48^, ^49^ The addition of immune cells may be a method for modelling acute inflammatory response triggered by MI.

The cardiac organoid model also allowed us to evaluate drug toxicity to cardiovascular system. Doxorubicin is a chemotherapy drug that has showed cardiotoxicity in hiPSC‐derived cardiomyocytes and endothelial cells, as well as different organoids.^6^, ^24^ Consistently, the hiPSC‐derived vcCOs we constructed accurately replicated drug‐induced toxicity in a dose‐ and time‐dependent manner. The comparative studies in vcCOs and 2D‐cultured cardiovascular cells might provide new insights into clinically relevant drug resistance.

In summary, we established a robust method for generation of vascularized and chambered complex vcCOs containing cardiomyocytes, endothelial cells, fibroblasts and among others, and verified their potential for cardiac disease modelling and drug evaluation. With the increased cellular composition and architectural complexity, we believed that hiPSC‐derived vcCOs will become more accurate models for cardiac pathophysiology research and drug development, and provide original transplant donors for clinical tissue/organ repair in future.

## AUTHOR CONTRIBUTIONS

S. Hu, W. Lei, Z. Shen and C. Zhang conceived and oversaw the project and co‐wrote the article. J. [Jingsi] Yang and W. Lei designed and conducted the experiment, analysed the data and wrote the article. Y. Xiao and Y. Lin performed a bioinformatics analysis to provide scRNA‐seq data. S. Tan, J. [Jiani] Yang, Z. Yang and D. Zhao conducted part of the experiment and co‐wrote the paper.

## FUNDING INFORMATION

This work was funded by the National Key R&D Program of China (2022YFA1104300, 2021YFA1101902), the National Natural Science Foundation of China (82241202, 82170364, 81970223), the Natural Science Foundation of Jiangsu Province (BK20201409), National Center for International Research (2017B01012) and Jiangsu Cardiovascular Medicine Innovation Center (CXZX202210).

## CONFLICT OF INTEREST STATEMENT

The authors have no conflicts of interest to disclose.

## Supporting information


**Figure S1.** Identification of smooth muscle cells and pericytes in vaschamcardioids. (A, B) Representative images of immunofluorescence staining for markers of the smooth muscle cell (α‐SMA, red), pericyte (PDGFRβ, red) and endothelial cell (PECAM1, green). Nuclei were stained with Hoechst 33342 (blue). Scale bar indicates 50 μm.
**Figure S2.** Flow cytometric analysis of the cellular components in vaschamcardioids. (A) cTnT‐positive cardiomyocytes (*n* = 3). (B) PECAM1‐positive endotheliocytes (*n* = 3). (C) COL1A2‐positice fibroblasts (*n* = 3).
**Figure S3.** Gene ontology analyses of genes expressed in the cardiomyocyte and endotheliocyte clusters. (A) Top 10 enriched cellular components (orange) and molecular functions (blue) for genes expressed in the cardiomyocyte cluster. (B) Top 10 cellular components (orange) and molecular functions (blue) for genes expressed in the endotheliocyte cluster. An FDR‐adjusted *p*‐value of 0.05 was set as a threshold.
**Figure S4.** Comparitive analysis of cardiomyocytes in vaschamcardioids and human adult/fetal hearts. UMAP_2 diagram indicated the integrated, dimensionality‐reduced clustering of cardiomyocytes in vaschamcardioids (vcCOs), human adult (date source: GSE109816) and fetal (data source: GSE106118) hearts.
**Figure S5.** Venn diagram of the ligand–receptor pairs between cardiomyocyte and fibroblast. Venn diagram showing overlap of ligand–receptor pairs between the cardiomyocyte‐to‐fibroblast (CM/FB) and fibroblast‐to‐cardiomyocyte (FB/CM) orientations.
**Figure S6.** Evaluation of cryo‐induced cardiac damage in cardiomyocyte spheres. (A) Masson's trichrome staining (blue, connective tissue; red, muscle) of cardiomyocyte spheres in the control (Ctrl), cryoinjury (Cryo) and cryoinjury plus captopril treatment (Cryo+CAP) groups. Masson staining was performed 3 days post‐injury. Scale bar, 100 μm. (B) Quantification of fibrotic areas in panel A. (C) Evaluation of the level of cTnT in culture medium by ELISA (*n* = 5). **p < 0.01, ***p < 0.001, and ns, not significant.
**Table S1.** Antibodies used for immunofluorescence (IF) and flow cytometry (FC).
**Table S2.** List of quantitative real‐time PCR primers.

**Movie S1.** Representative movie of beating vaschamcardioids at day 15.

**Movie S2.** Representative movie of beating vaschamcardioids after culture for 8 weeks.

**Movie S3.** Representative movie of control vaschamcardioids in the cryoinjury section.

**Movie S4.** Representative movie of cryoinjury vaschamcardioids.

**Movie S5.** Representative movie of cryoinjury vaschamcardioids after captopril treatment.

## Data Availability

All data generated or analysed during this study are available from the corresponding author upon reasonable request.
