# Supplementary material for: Generation of human vascularized and chambered cardiac organoids for cardiac disease modelling and drug evaluation
Source: Cell Prolif. 2024 Mar 7;57(8):e13631. doi: 10.1111/cpr.13631 (PMC11294415; doi:10.1111/cpr.13631)
Supplement: Supplementary file 1 — Figure S1. Identification of smooth muscle cells and pericytes in vaschamcardioids. (A, B) Representative images of immunofluorescence staining for markers of the smooth muscle cell (α‐SMA, red), pericyte (PDGFRβ, red) and endothelial cell (PECAM1, green). Nuclei were stained with Hoechst 33342 (blue). Scale bar indicates 50 μm. Figure S2. Flow cytometric analysis of the cellular components in vaschamcardioids. (A) cTnT‐positive cardiomyocytes (n = 3). (B) PECAM1‐positive endotheliocytes (n = 3). (C) COL1A2‐positice fibroblasts (n = 3). Figure S3. Gene ontology analyses of genes expressed in the cardiomyocyte and endotheliocyte clusters. (A) Top 10 enriched cellular components (orange) and molecular functions (blue) for genes expressed in the cardiomyocyte cluster. (B) Top 10 cellular components (orange) and molecular functions (blue) for genes expressed in the endotheliocyte cluster. An FDR‐adjusted p‐value of 0.05 was set as a threshold. Figure S4. Comparitive analysis of cardiomyocytes in vaschamcardioids and human adult/fetal hearts. UMAP_2 diagram indicated the integrated, dimensionality‐reduced clustering of cardiomyocytes in vaschamcardioids (vcCOs), human adult (date source: GSE109816) and fetal (data source: GSE106118) hearts. Figure S5. Venn diagram of the ligand–receptor pairs between cardiomyocyte and fibroblast. Venn diagram showing overlap of ligand–receptor pairs between the cardiomyocyte‐to‐fibroblast (CM/FB) and fibroblast‐to‐cardiomyocyte (FB/CM) orientations. Figure S6. Evaluation of cryo‐induced cardiac damage in cardiomyocyte spheres. (A) Masson's trichrome staining (blue, connective tissue; red, muscle) of cardiomyocyte spheres in the control (Ctrl), cryoinjury (Cryo) and cryoinjury plus captopril treatment (Cryo+CAP) groups. Masson staining was performed 3 days post‐injury. Scale bar, 100 μm. (B) Quantification of fibrotic areas in panel A. (C) Evaluation of the level of cTnT in culture medium by ELISA (n = 5). **p < 0.01, ***p < 0.001, and ns [file CPR-57-e13631-s002.docx]

**SUPPLEMENTAL INFORMATION**

**Generation of human vascularized and chambered cardiac organoids for cardiac disease modeling and drug evaluation**

Jingsi Yang^1, #^, Wei Lei^1, #^, Yang Xiao^1^, Shuai Tan^1^, Jiani Yang^1^, Yingjiong Lin^1^, Zhuangzhuang Yang^1^, Dandan Zhao^1^, Chunxiang Zhang^2, *^, Zhenya Shen^1, *^, Shijun Hu^1, *^

^1^Department of Cardiovascular Surgery of the First Affiliated Hospital & Institute for Cardiovascular Science, Collaborative Innovation Center of Hematology, State Key Laboratory of Radiation Medicine and Protection, Suzhou Medical College, Soochow University, Suzhou 215000, China; ^2^Department of Cardiology, Key Laboratory of Medical Electrophysiology, Ministry of Education, Institute of Cardiovascular Research, the Affiliated Hospital, Southwest Medical University, Luzhou 646000, China.

^#^ These authors contributed equally to this work

^*^ To whom correspondence should be addressed, Shijun Hu, Email: [shijunhu@suda.edu.cn](mailto:shijunhu@suda.edu.cn); Zhenya Shen, [uuzyshen@aliyun.com](mailto:uuzyshen@aliyun.com); Chunxiang Zhang, [zhangchx999@163.com](mailto:zhangchx999@163.com)


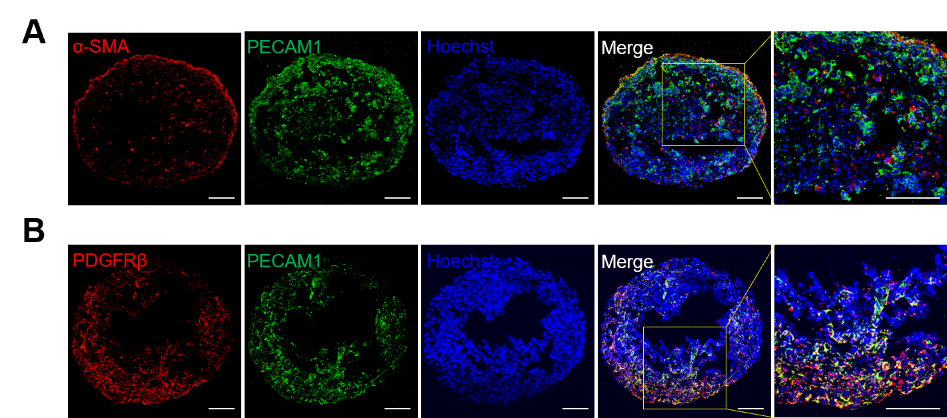


**Supplemental Figure 1. Identification of smooth muscle cells and pericytes in vcCOs.** (A-B) Representative images of immunofluorescence staining for markers of the smooth muscle cell (α-SMA, red), pericyte (PDGFRβ, red) and endothelial cell (PECAM1, green). Nuclei were stained with Hoechst 33342 (blue). Scale bar indicates 50 μm.


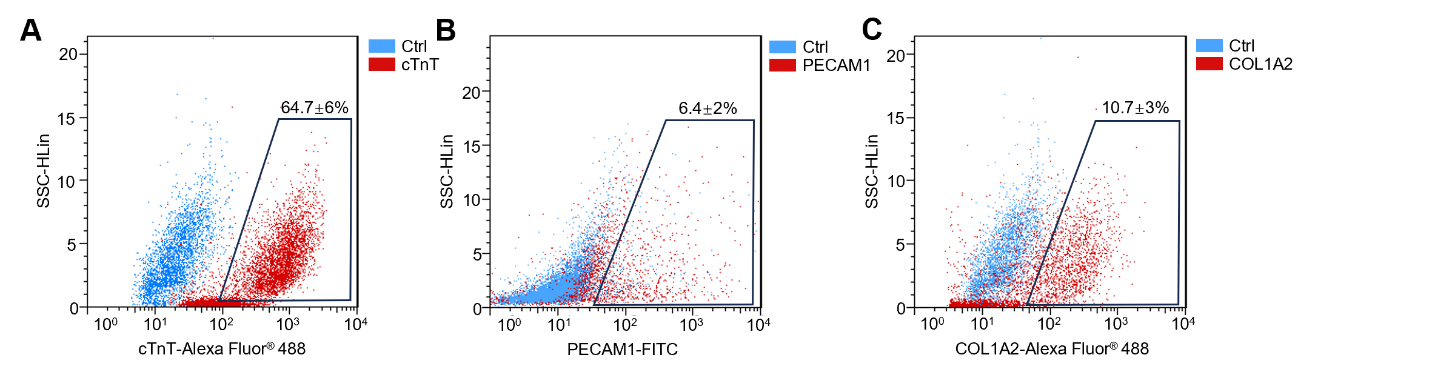


**Supplemental Figure 2. Flow cytometric analysis of the cellular components in vcCOs.** (A) cTnT-positive cardiomyocytes (n=3). (B) PECAM1-positive endotheliocytes (n=3). (C) COL1A2-positice fibroblasts (n=3).


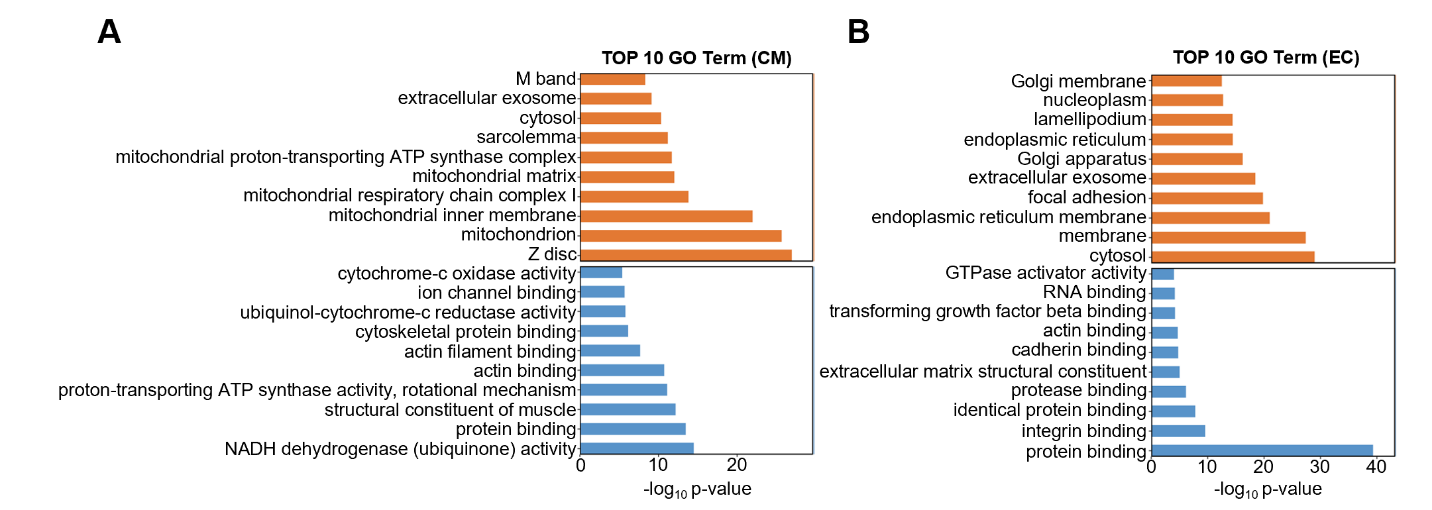


**Supplemental Figure 3. Gene Ontology analyses of genes expressed in the cardiomyocyte and endotheliocyte clusters.** (A) Top 10 enriched cellular components (orange) and molecular functions (blue) for genes expressed in the cardiomyocyte cluster. (B) Top 10 cellular components (orange) and molecular functions (blue) for genes expressed in the endotheliocyte cluster. An FDR-adjusted p-value of 0.05 was set as a threshold.


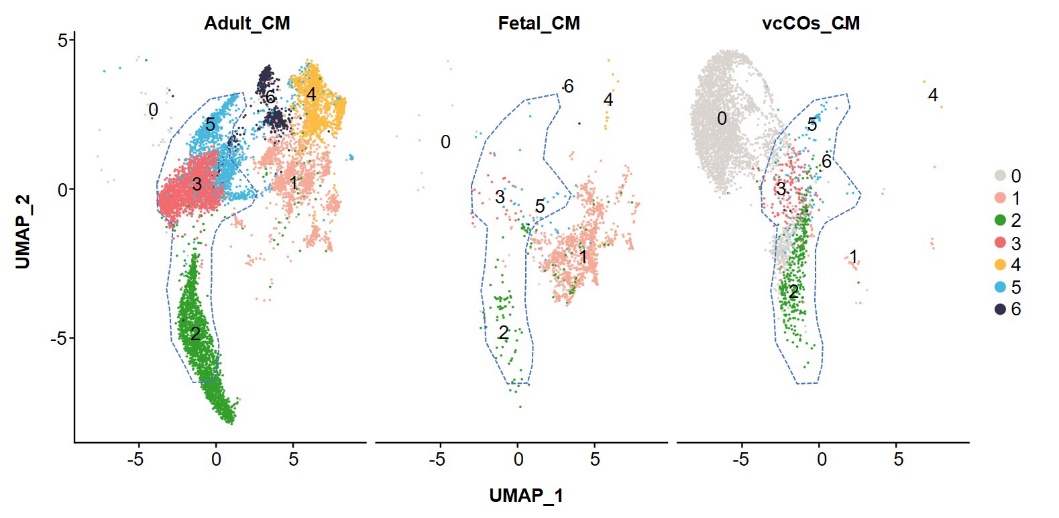


**Supplemental Figure 4. Comparitive analysis of cardiomyocytes in vcCOs and human adult/fetal hearts.** UMAP_2 diagram indicated the integrated, dimensionality-reduced clustering of cardiomyocytes in vcCOs, human adult (date source: GSE109816) and fetal (data source: GSE106118) hearts.


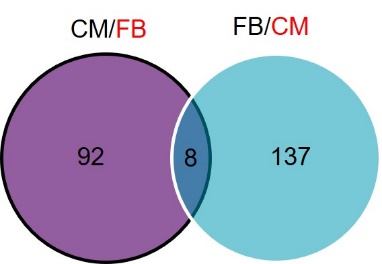


**Supplemental Figure 5. Venn diagram of the ligand-receptor pairs between cardiomyocyte and fibroblast.** Venn diagram showing overlap of ligand-receptor pairs between the cardiomyocyte-to-fibroblast (CM/FB) and fibroblast-to-cardiomyocyte (FB/CM) orientations.


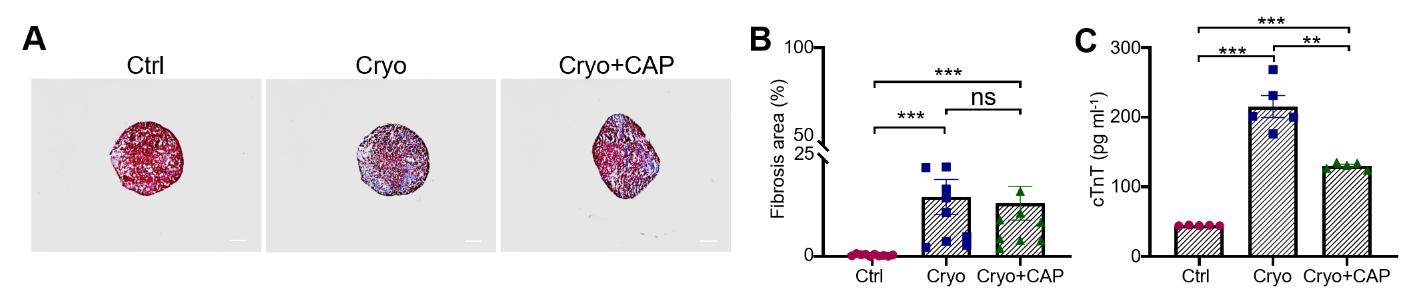


**Supplemental Figure 6. Evaluation of cryo-induced cardiac damage in cardiomyocyte spheres.** (A) Masson’s trichrome staining (blue, connective tissue; red, muscle) of cardiomyocyte spheres in the control (Ctrl), cryoinjury (Cryo), and cryoinjury plus captopril treatment (Cryo+CAP) groups. Masson staining was performed 3 days post injury. Scale bar, 100 μm. (B) Quantification of fibrotic areas in panel A. (C) Evaluation of the level of cTnT in culture medium by ELISA (n=5). **p < 0.01, ***p < 0.001, and ns, not significant.

**Supplemental table 1. Antibodies used for immunofluorescence (IF & FC).**

| **Antibody** | **Company** | **Catalog Number** | **IF** |
| --- | --- | --- | --- |
| Cardiac troponin T (cTnT) | Proteintech | 15513-1-AP | 1:200 |
| PECAM1 | Proteintech | 66065-2-IG | 1:200 |
| Sarcomeric Alpha Actinin  (α-actinin) | Abcam | Ab9465 | 1:200 |
| Smooth muscle actin (α-SMA) | Proteintech | 14395-1-AP | 1:200 |
| PDGFRβ | Proteintech | 13449-1-AP | 1:200 |
| Collagen Type I (COL1A2) | Proteintech | 67288-1-IG | 1:200 |
| PECAM1-FITC | Invitrogen | 1926759 | 1:100 |
| Alexa Fluor® 488 AffiniPure Donkey Anti-Mouse IgG (H+L) | Jackson ImmuoResearch | 715-545-151 | 1:500 |
| Alexa Fluor® 488 AffiniPure Donkey Anti-Rabbit IgG (H+L) | Jackson ImmuoResearch | 711-545-152 | 1:500 |
| Alexa Fluor® 647 AffiniPure Donkey Anti-Rabbit IgG (H+L) | Jackson ImmunoResearch | 711-605-152 | 1:500 |
| Alexa Fluor® 594 AffiniPure Donkey Anti-Mouse IgG (H+L) | Jackson ImmunoResearch | 715-585-151 | 1:500 |

**Supplemental table 2. List of quantitative real-time PCR primers.**

| **Gene Name** | **Sense (5’ to 3’)** | **Antisense (5’ to 3’)** |
| --- | --- | --- |
| *TNNT2* | ACCAAAGCCCAGGTCGTTC | CAGCGCCTGCAACTCATTC |
| *MYH7* | ACCTGTCCAAGTTCCGCAAG | TCATTCAAGCCCTTCGTGCC |
| *MYH6* | CAAGAGCCGTGACATTGGTG | AGGTTGGCAAGAGTGAGGTT |
| *PECAM1* | AACAGCAAAAGCCACAAAGACG | GGGGTCAGAGGAAGAGATAAAGTTG |
| *CDH5* | TCAAGCACACTGATCCCGTC | GCTACCACCACTCCCATAGC |
| *CD34* | CAAGCCACCAGAGCTATTCC | TAGCCAGTGATGCCCAAGAC |
| *α-SMA* | TCCGGAAACAGTATGAAGACCA | CACACAACGCTGGCAATTCA |
| *VIM* | AAGCTGCGTTTCGAGGTCTT | GTCTCCTTGTTGCCCTTGGT |
| *18S rRNA* | GTAACCCGTTGAACCCCATT | CCATCCAATCGGTAGTAGCG |

**Supplemental movie 1. Representative movie of beating vcCOs at day 15.**

**Supplemental movie 2. Representative movie of beating vcCOs after culture for 8 weeks**

**Supplemental movie 3. Representative movie of control vcCOs in the cryoinjury section.**

**Supplemental movie 4. Representative movie of** **cryoinjury vcCOs.**

**Supplemental movie 5. Representative movie of cryoinjury** **vcCOs after captopril treatment.**
